# Strayfield calculation for micromagnetic simulations using true periodic boundary conditions

**DOI:** 10.1038/s41598-021-88541-9

**Published:** 2021-04-28

**Authors:** Florian Bruckner, Amil Ducevic, Paul Heistracher, Claas Abert, Dieter Suess

**Affiliations:** Faculty of Physics, University of Vienna, Vienna, Austria

**Keywords:** Computational methods, Magnetic properties and materials

## Abstract

We present methods for calculating the strayfield in finite element and finite difference micromagnetic simulations using true periodic boundary conditions. In contrast to pseudo periodic boundary conditions, which are widely used in micromagnetic codes, the presented methods eliminate the shape anisotropy originating from the outer boundary. This is a crucial feature when studying the influence of the microstructure on the performance of composite materials, which is demonstrated by hysteresis calculations of soft magnetic structures that are operated in a closed magnetic loop configuration. The applied differential formulation is perfectly suited for the application of true periodic boundary conditions. The finite difference equations can be solved by a highly efficient Fast Fourier Transform method.

## Introduction

Micromagnetic simulation are often used for the characterization of magnetic materials with a certain microstructure. Since the magnetic samples are very large, only a small part of the material can be simulated. A naive truncation of the magnetic domain would lead to strong shape anisotropy originating from surface effects. Periodic boundary conditions (PBCs) allow to eliminate this influence of the surface by modeling periodic images of the primary supercell.

Most well-known micromagnetic finite difference simulation packages like OOMMF^1^, MuMax$^3$^2^, magnum.fd^3^, magnum.af^4^ and Fidimag^5^ calculate the demagnetization field without PBCs using an analytic expression of the demagnetization tensor of homogeneously magnetized cubes^6^ combined with an efficient FFT method making use of the convolution theorem^7^. Since this method is based on an integral formulation of the magnetic strayfield equations, incorporating PBCs requires the summation over an infinite sum of periodic images. Solutions have been proposed for 1D and 2D problems^8,9^, however an extension to 3D is not possible since the occurring sums are not absolutely convergent. Using point-dipoles instead of finite magnetized cubes seems to overcome this limitation and allows true 3D periodic boundary conditions^10^. In contrast to true PBCs which require an infinite summation, some codes use pseudo PBCs where the summation is truncated after a finite number of periodic images^2,3^. Apart from the easier implementation those methods are well suited for systems of intermediate size, where a finite number of periodic images is sufficient to model the complete sample. This principle has even been applied to FEM simulation where it is called *macro-geometry*^11^. Since finite element calculations are based on the differential form of the strayfield equation (real) PBCs can be directly applied by providing a proper cell-connectivity which represents the periodic structure.

We propose the application of PBCs for 3D problems based on a differential form of the strayfield equations both for finite elements (FE) and finite differences (FD). We focus on the efficient strayfield calculation because it is the most time-consuming part of micromagnetic simulations. Due to the long-range interaction the strayfield is usually solved using integral formulations. Since we use a differential formulation, the discretization using FE or FD with PBCs is straightforward, however it requires the solution of a sparse system of equations. In case of FD the use of a Fourier space method allows direct inversion of the system and offers a significant speed up.

## Strayfield calculation using PBCs

The magnetic strayfield $\varvec{h}$ of a given magnetization $\varvec{m}$ can be calculated by means of magnetostatic Maxwell’s equations. Since the magnetic strayfield is curl-free a scalar potential formulation can be used:

1 $$\begin{aligned} \Delta u = {{\,\text{div}\,}}\varvec{m}, \end{aligned}$$

where *u* is the magnetic scalar potential and the magnetic field can be calculated as $\varvec{h} = -\nabla u$. Proper boundary conditions need to be defined in order to obtain a unique solution.

If the magnetization is localized in a magnetic region $\Omega \subset {\mathbb{R}}^3$ the problem is called an open-boundary problem with $u = {\mathcal {O}}({\frac{1}{r}})$ as $r \rightarrow \infty$. Since the boundary condition are not known at the surface of the magnet, direct use of the differential formulation (1) would require the discretization of an (infinite) air domain outside of the magnet. Accurate and efficient methods for solving open-boundary problems are often based on a corresponding integral formulation. In finite-element micromagnetics, the strayfield is usually solved in the differential form (1) and a hybrid method by Fredkin and Koehler^12^ is used in order to resolve the boundary conditions on the magnetic surface by means of the boundary element method. In finite-difference micromagnetics, the direct integration of the strayfield tensor^6^ combined with an efficient FFT based convolution is commonly used.

**Figure 1:**
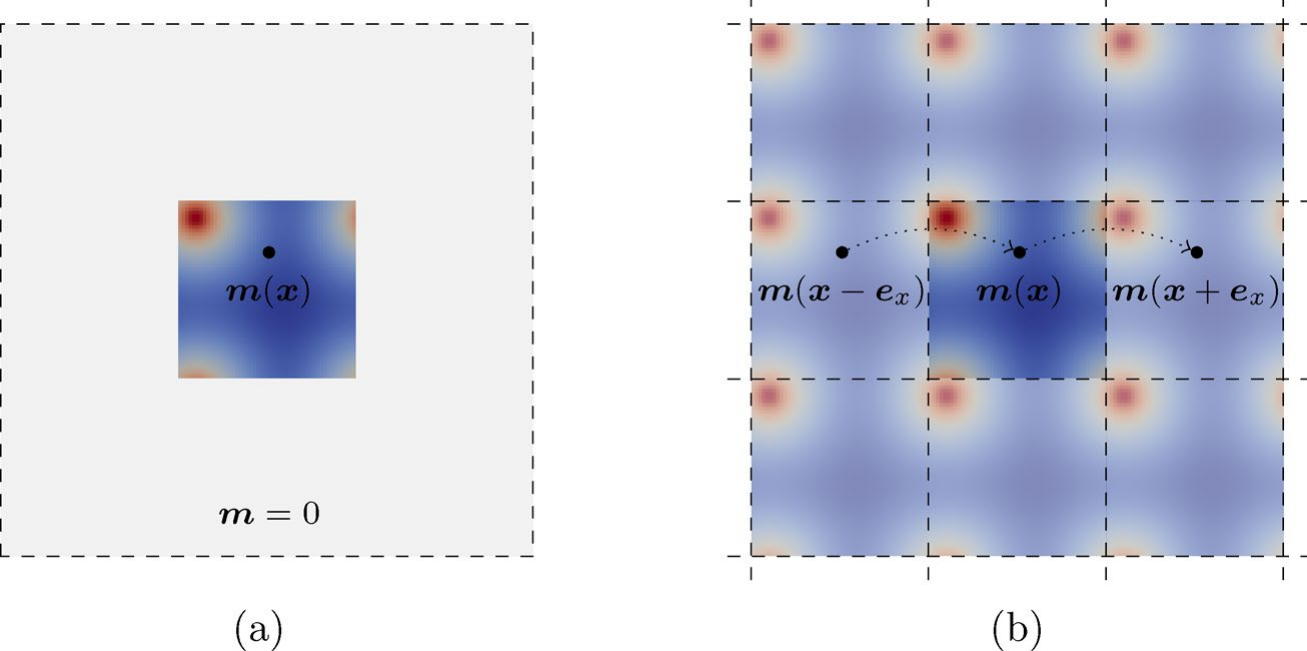
Magnetization configurations in case of (**a**) open-boundary conditions and (**b**) periodic boundary conditions.

When dealing with large periodic structures, only a fraction of the magnetic sample can be discretized. Assuming open-boundary conditions is no longer valid in this case. Instead, a periodic magnetization can be assumed (see Fig. 1) and thus PBCs can be used as proper boundary conditions for *u*.

### Finite element discretization

The finite element formulation is based on the weak form of the differential equation (1)

2 $$\begin{aligned} \int _\Omega \nabla u \cdot \nabla \phi _i - \oint _{\partial \Omega } \partial _{\mathbf {n}} u \; \phi _i = \int _\Omega {\mathbf {m}} \cdot \nabla \phi _i - \oint _{\partial \Omega } {\mathbf {m}} \cdot {\mathbf {n}} \; \phi _i, \end{aligned}$$

with proper test functions $\phi _i$. $\partial \Omega$ would be the domain boundary with a corresponding unit normal ${\mathbf {n}}$. In case of PBCs those surface intergrals vanish, because there is no physical domain boundary. For the application of PBCs a mapping of boundary nodes to their periodic images needs to be provided in order to eliminate the corresponding degree of freedoms. We used finite element packages *FEniCS*^13^ or *firedrake*^14^ (via *firedrake-periodicity*), which offer capabilities to define PBCs.

**Figure 2:**
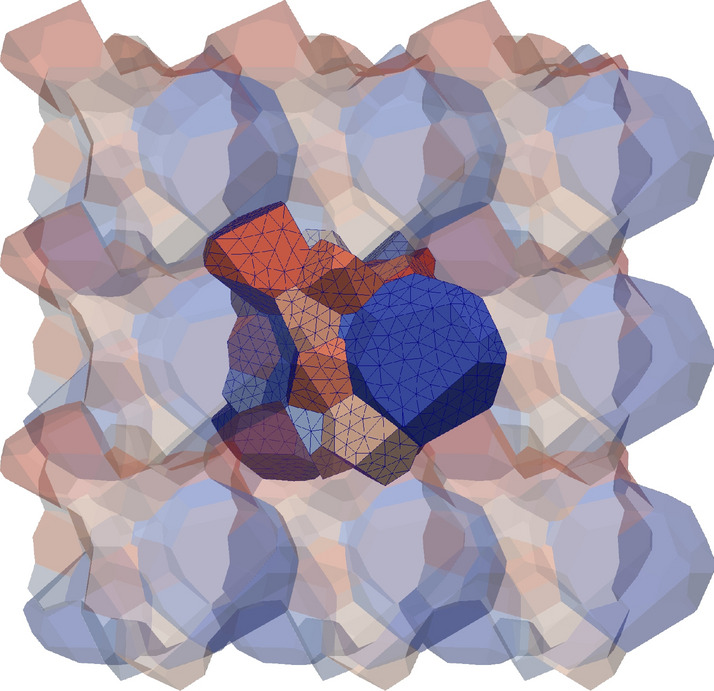
Periodic grain structure with periodic FEM mesh created with *neper*.

One difficulty when dealing with PBCs in FEM is the creation of a periodic mesh. Sophisticated periodic grain structures can be created with *neper* (see for example Fig. 2). Compared with a finite difference discretization the finite element model provides a better geometry representation, but requires the solution of an (unstructured) sparse system of equations, which significantly increases execution time by some orders of magnitude.

### Finite difference discretization

The finite difference method is based on a rectangular $N_x \times N_y \times N_z$ mesh. In order to use an efficient FFT method for the solution of the occurring system of equations, we assume that the mesh is equidistant. Thus the index set (*i*, *j*, *k*) is sufficient to identify each vertex of the mesh.

3 $$\begin{aligned} \varvec{x}_{i,j,k} = \begin{pmatrix} x_i \\ x_j \\ x_k\end{pmatrix} = \begin{pmatrix} x_0 + i \; \Delta x \\ y_0 + j \; \Delta y \\ z_0 + k \; \Delta z \end{pmatrix} = \varvec{x}_0 + \Delta \varvec{x} \quad \text {with} \quad \begin{matrix} i = 0 \ldots N_x-1 \\ j = 0 \ldots N_y-1 \\ k = 0 \ldots N_z-1 \end{matrix} \end{aligned}$$

**Figure 3:**
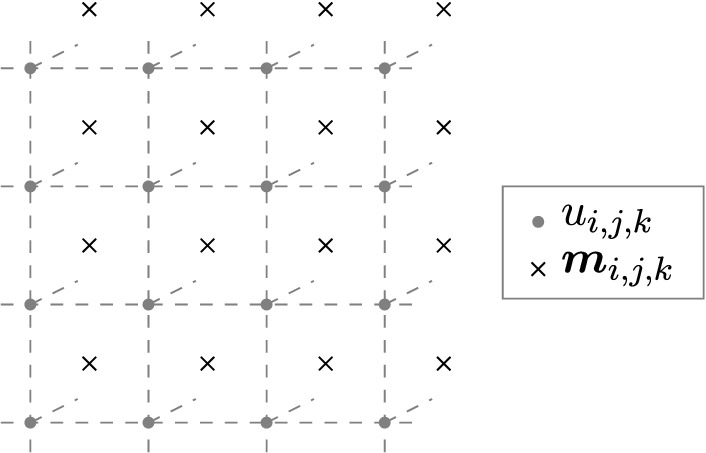
Finite difference grid used for the discretization of *u* and ${{\,\text{div}\,}}(\varvec{m})$ (on vertices) as well as $\varvec{m}$ and $\varvec{h}$ (on cell centers).

In the following, we use the convention that *u* and ${{\,\text{div}\,}}(\varvec{m})$ are defined on the grid vertices, whereas $\varvec{m}$ and $\varvec{h}$ are defined at the cell centers (see Fig. 3). In the case of PBCs, the number of grid points and cell centers are equal and the cell centers form a shifted grid.

$$\begin{aligned} u_{i,j,k}&= u(\varvec{x}_{i,j,k}) \\ ({{\,\text{div}\,}}\varvec{m})_{i,j,k}&= ({{\,\text{div}\,}}\varvec{m})(\varvec{x}_{i,j,k}) \\ \varvec{m}_{i,j,k}&= \varvec{m}(\varvec{x}_{i,j,k} + \Delta \varvec{x}/2) \\ \varvec{h}_{i,j,k}&= \varvec{h}(\varvec{x}_{i,j,k} + \Delta \varvec{x}/2) \end{aligned}$$

The chosen convention allows to use central differences and leads to the following discrete approximations of the continuous differential operators

4 $$\begin{aligned} {} & \begin{matrix} (\Delta u)_{i,j,k} \; \approx \; &{}{\frac{u_{i+1,j,k} - 2 u_{i,j,k} + u_{i-1,j,k}}{\Delta x^2}} \\ &{}+\frac{u_{i,j+1,k} - 2 u_{i,j,k} + u_{i,j-1,k}}{\Delta y^2} \\ &{}+\frac{u_{i,j,k+1} - 2 u_{i,j,k} + u_{i,j,k-1}}{\Delta z^2}, \end{matrix}\nonumber \\&\begin{matrix} ({{\,\text{div}\,}}\varvec{m})_{i,j,k} \; \approx \; &{}{\frac{m^x_{i,j,k} - m^x_{i-1,j,k}}{\Delta x}} \\ &{}+\frac{m^y_{i,j,k} - m^y_{i,j-1,k}}{\Delta y} \\ &{}+\frac{m^z_{i,j,k} - m^z_{i,j,k-1}}{\Delta z}, \end{matrix}\nonumber \\&(\nabla \varvec{u})_{i,j,k} \; \approx \; \begin{pmatrix} {\frac{u_{i+1,j,k} - u_{i,j,k}}{\Delta x}} \\ {\frac{u_{i,j+1,k} - u_{i,j,k}}{\Delta y}} \\ {\frac{u_{i,j,k+1} - u_{i,j,k}}{\Delta z}} \end{pmatrix}. \end{aligned}$$

Using these sparse discrete operators allows to solve the discrete version of Eq. (1)

5 $$\begin{aligned} (\Delta u)_{i,j,k} = ({{\,\text{div}\,}}\varvec{m})_{i,j,k}. \end{aligned}$$

### Efficient implementation using a fourier space method

The discrete system (5) can be solved directly, as it has to be done in the finite element method. Due to the regular (and equidistant) grid, the FD system can also be solved in Fourier space, where all differential operators become algebraic and the system can be directly inverted.

The potential $u_{i,j,k}$ can be represented by means of the corresponding Fourier-space potential $\tilde{u}_{i,j,k}$ using the Discrete Fourier Transform (DFT)6where  is the imaginary unit and the wave-vectors $k_l$, $k_m$, and $k_n$ are defined by

$$\begin{aligned} k_l = {\frac{2 \pi l}{N_x}}, \; k_m ={ \frac{2 \pi m}{N_y}}, \; k_n = {\frac{2 \pi n}{N_z}} \end{aligned}$$

A similar Ansatz is used for the other fields $\varvec{m}$, and $\varvec{h}$. When substituting the Fourier-space representation (6) into the definition of the discrete operators (4) the spatial indices *i*, *j*, *k* only occur within the exponent, which results in a simple multiplicative phase factor for all neighbouring cells. Simplifying all occurring prefactors finally yields7

One can see that the non-local terms within the operator lead to local pre-factors within Fourier-space. Using the fact that the Fourier basis functions are linearly independent of each other, allows to explicitly express the Fourier coefficients of the strayfield $\tilde{\varvec{h}}_{l,m,n}$ as a function of the Fourier coefficients of the magnetization $\tilde{\varvec{m}}_{l,m,n}$:
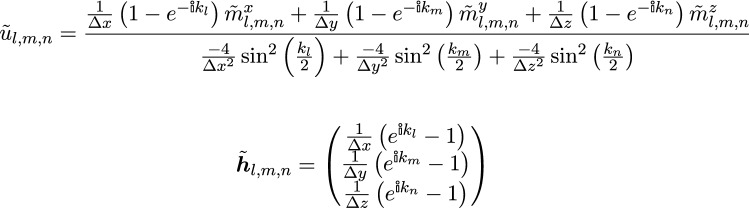


Note that evaluation of $\tilde{u}_{0,0,0}$, which represents the constant part of the potential, would lead to a division by 0. However it can be set to zero since it has no influence on the magnetic field.

Due to the use of the FFT, the resulting algorithm is very efficient. Compared with the non-periodic FFT strayfield calculation, which is based on the integral formulation, the assembly and storage of the demagnetization tensor can be avoided. Since no zero-padding is necessary the system size gets even smaller in case of PBCs. As a further optimization one can use a real-FFT, since the input magnetization as well as the resulting strayfield is purely real-valued. This leads to a further speedup by a factor of 2, as well as to a reduced storage size. The presented method is well suited for parallel execution on modern GPUs.

The FD method has been implemented within the micromagnetic code magnum.af and can be used on CPU or GPU, respectively. The FE method utilizing true PBCs has been implemented using magnum.pi. Table 1 summarizes timings of the strayfield calculation using the presented methods.

**Table 1:** Timing comparison for the FFT strayfield calculation on a cubic $N \times N \times N$ grid.

*N*	$N^3$	$t^{\text {fd}}_{\text {cpu}}$ (s)	$t^{\text {fd}}_{\text {gpu}}$ (s)	$t^{\text {fem}}_{\text {cpu}}$ (s)
10	1000	0.0013	0.0014	0.005
50	125,000	0.0361	0.0015	1.310
100	1000,000	0.2660	0.0022	17.33
160	4096,000	1.3463	0.0088	–
220	10648,000	4.2463	0.0166	–
260	17576,000	9.6362	0.0547	–

$t^{\text {fd}}_{\text {cpu}}$ (s) was calculated on a Quad-Core Intel i7-5600U CPU @ 2.60GHz using Numpy’s real-FFT transformation, while $t^{\text {fd}}_{\text {gpu}}$ (s) was calculated on a Tesla V100 GPU using OpenCL. $t^{\text {fem}}_{\text {cpu}}$ shows the timings of a FE discretization solved on the same CPU using a standard *gmres*/*ilu* method.

## Numerical experiments

The presented method is validated by comparison with analytical calculations. The trivial case of a homogeneously magnetized bulk material leads to zero demagnetization field according to Eq. ((1)) since ${{\,\text{div}\,}}{\mathbf {m}} = 0$ everywhere.

A non-trivial solution can be found for an infinite number of infinitely extended thin-films with thickness $d_1$ and a spacing between each thin-film of $d_0$. Each thin-film is magnetized perpendicular to the film plane (in $-x$ direction) with a saturation magnetization of $M_s$. Due to symmetry considerations the resulting field only points in the perpendicular direction, which reduces the problem to one dimension. Since the magnetization is constant inside of the thin-film and zero outside, one can again assume ${{\,\text{div}\,}}{\mathbf {m}} = 0$ in both regions. Thus the resulting potential is a piecewise linear function $u(x) = a_i x + b_i \; {\text {for}} \; x \in \Omega _i$, where $i \in (0,1)$ denotes the domain index.

Considering the jump-conditions of the magnetic scalar potential

$$\begin{aligned}{}[[ u ]]&= 0 \\ \left[ \left[ \frac{\partial u}{\partial {\mathbf {n}}} \right] \right]&= {\mathbf {M}} \cdot {\mathbf {n}} \end{aligned}$$

leads to $a_0 - a_1 = M_s$, where [[.]] denotes the jump across the domain boundary. Periodicity and continuity of the potential furthermore yields $a_1 d_1 + a_0 d_0 = 0$, where $d_0$ and $d_1$ are the thickness of the thin-film and the spacing layer, respectively. Putting everything together yields

$$\begin{aligned} a_0 = \frac{M_s}{1+d_0 / d_1}&a_1 = -\frac{M_s}{1+d_1 / d_0} \end{aligned}$$

**Figure 4:**
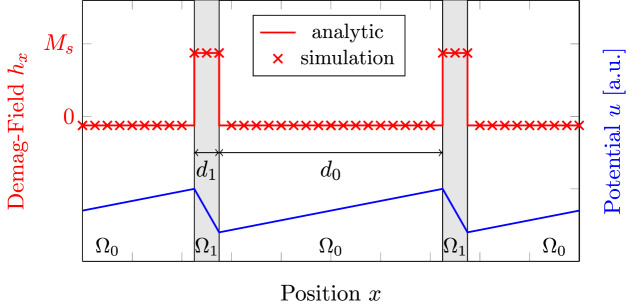
Comparison of the proposed FD method with analytical results for an infinite number of infinitely extended thin-films.

Note that in the limit $d_0 \rightarrow \infty$ one ends up with the well known result for a single infinite thin-film with a field $-M_s$ inside of the film and 0 outside. It is remarkable that while a single thin-film shows no external field, an infinite number of films do. The calculated fields and the corresponding potential is visualized in Fig. 4 and compared with simulation results using the presented FD method.

**Figure 5:**
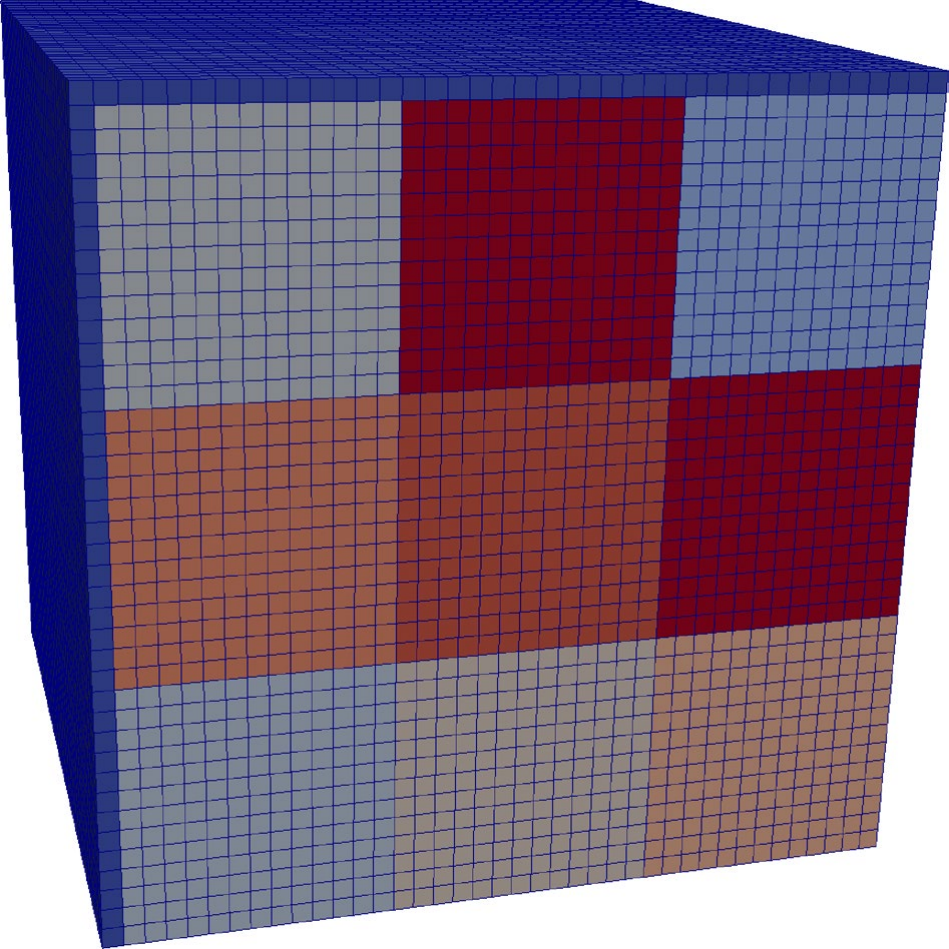
Simplified geometry of the SMC material consisting of $3 \times 3 \times 3$ magnetic grains separated with one non-magnetic interparticle layer (dark blue). The size of each grain is ${300}\,{\text {nm}} \times {300}\,{\text {nm}} \times {300}\,{\text {nm}}$, whereas the interparticle thickness is varied.

The performance of the presented method should be further demonstrated by the calculation of the hysteresis loop of a soft-magnetic-composite (SMC) material. The material consists of isolated particles, with each particle itself consisting of several magnetic grains. The simulation is restricted to a primary cell containing only one magnetic particle, consisting of $3 \times 3 \times 3$ grains with a size of ${300}\,{\text{ nm }} \times {300}\,{\text{ nm }} \times {300}\,{\text{ nm }}$, as well as a non-magnetic interparticle layer (see Fig. 5). PBCs are used to mimic interparticle interactions and to avoid surface effects. The width of the interparticle layer $w_{\text {gap}}$ is varied and its influence on the magnetic hysteresis is studied.

For the FE model a (non-equidistant) regular mesh is used. Each dimension is divided into $N = 3 N_i + 1$ parts, where $N_i$ is the number of divisions of each grain. The grid spacing within the grains is constant, whereas the thickness of the interparticle layer can be adjusted as desired. In contrast, the FD discretization requires using an equidistant grid, which limits the possible interparticle thicknesses to integer divisors of the grain-size. Furthermore many FFT libraries require that largest prime factor of the system size is smaller than a certain value. This is based on the fact, that the FFT performs best for system sizes $N = 2^M$ for integer *M*. Performance decreases dramatically (more than one order of magnitude) if system sizes with much higher prime-factors are used^2^. This general limitation of the FD method also results in very large system sizes for small width of the inter-particle layer and makes the more flexible FE method still competitive.

**Figure 6:**
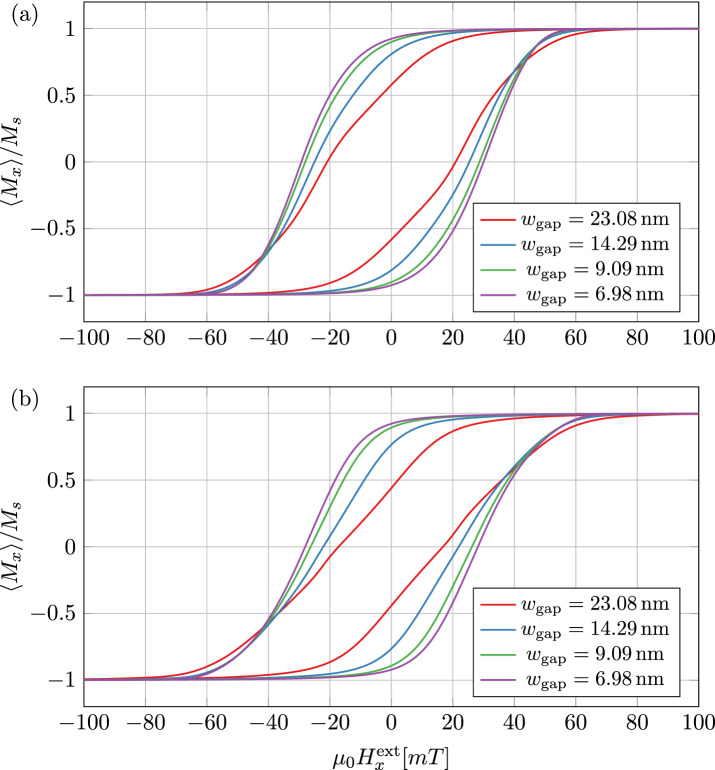
Hysteresis curves of the SMC material for varying interparticle widths $w_{\text {gap}}$ using true PBCs implemented with the (**a**) FE method and (**b**) FD method.

**Figure 7:**
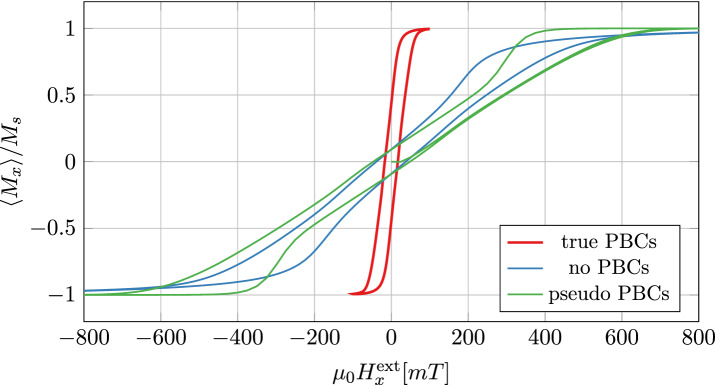
Finite difference hysteresis curves of the SMC material for interparticle widths $w_{\text {gap}} = {23.08}\,{\text {nm}}$ with different boundary conditions. Without true PBCs the external field range has to be extended to $\pm {2.5}\,{\text {T}}$, in order to fully saturate the material.

The used material parameters can be found in Table 2. The magnetic anisotropy axes within the 27 grains are randomly distributed. A homogeneous external magnetic field is applied and linearly varied from $-100\,{\text{ mT }} \, \text{to} \, 100\,{\text{ mT }}$ with a frequency of ${100}\,{\text{ MHz }}$. The resulting hysteresis loops for FE and FD method can be found in Fig. 6. It can be seen that a larger interparticle layer leads to a smaller hysteresis and thus reduces the hysteresis losses of the material. The dramatic influence of self-demagnetization without using true PBCs is demonstrated in Fig. 7. Due to the strong demagnetization effects, the external field range needs to be extended to $-2.5\,{\text{ T }} \, \text{to} \ 2.5\,{\text{ T }}$. Without PBCs the subtle effect of varying interparticle layer widths is superimposed by a much stronger finite size-effect, which additionally depends on the shape of the boundary. Since the influence of the boundary can be subtracted out only in average, extracting the desired macroscopic material properties will be much harder and less accurate.

The FE and FD simulations with true PBCs have been performed with magnum.pi, or magnum.af, respectively. The FD simulations without PBCs have been performed with magnum.af and validated with MuMax$^3$. The FD simulations using pseudo PBCs have been performed with MuMax$^3$.

**Table 2:** Micromagnetic material parameters used within the magnetic grains as well as for the non-magnetic interparticle layer.

	Magnetic grains	Interparticle layer
**Magnetic polarization**		
$J_s\,({\text{ T }})$	1.5	0.0001
**Exchange constant**		
$A\,({\text {J/m}})$	$10\times 10^{-12}$	0.0
**Uniaxial anisotropy constant**		
$K_u\,({\text{ J/m }}^{3})$	$8 \times 10^{3}$	0.0
**Damping constant**		
$\alpha$	1.00	1.00

The easy axes of the uniaxial anisotropy are randomly distributed.

## Conclusion

The importance of using true PBCs for the calculation of material properties without the influence of surface effects has been pointed out. An efficient FFT-based FD strayfield calculation providing true 3D PBCs has been presented. This method perfectly complements methods for 1D and 2D periodic boundary conditions. Those methods are based on integral formulation and cannot be extended to the 3D case. The discretization using finite elements is straightforward and may provide benefits for more complicated geometries. Even for the provided test case it allows a more flexible choice of the interparticle width $w_{\text {gap}}$.

## References

[1] Donahue, M. J. & Porter, D. G. Oommf user's guide, version 1.0, Interagency Report NISTIR 6376, National Institute of Standard and Technology, Gaithersburg, MD (Sept 1999). http://math.nist.gov/oommf. (2010).

[2] Vansteenkiste A, Leliaert J, Dvornik M, Helsen M, Garcia-Sanchez F, Van Waeyenberge B (2014). The design and verification of mumax3. AIP Adv..

[3] C. Abert. magnum.fd—A finite-difference/fft package for the solution of dynamical micromagnetic problems. https://github.com/micromagnetics/magnum.fd. (2013).

[4] Heistracher P, Bruckner F, Abert C, Vogler C, Suess D (2020). Hybrid fft algorithm for fast demagnetization field calculations on nonequidistant magnetic layers. J. Magnet. Magnet. Mater..

[5] M.-A. Bisotti, D. Cortés-Ortuño, R. A. Pepper, W. Wang, M. Beg, T. Kluyver, & H. Fangohr. Fidimag—A finite difference atomistic and micromagnetic simulation package. arXiv preprint arXiv:2002.04318 (2020).

[6] Newell AJ, Williams W, Dunlop DJ (1993). A generalization of the demagnetizing tensor for nonuniform magnetization. J. Geophys. Res. Solid Earth.

[7] Abert C, Bruckner F, Vogler C, Windl R, Thanhoffer R, Suess D (2015). A full-edged micromagnetic code in fewer than 70 lines of numpy. J. Magn. Magn. Mater..

[8] Lebecki KM, Donahue MJ, Gutowski MW (2008). Periodic boundary conditions for demagnetization interactions in micromagnetic simulations. J. Phys. D Appl. Phys..

[9] Wang W, Mu C, Zhang B, Liu Q, Wang J, Xue D (2010). Twodimensional periodic boundary conditions for demagnetization interactions in micromagnetics. Comput. Mater. Sci..

[10] Wysocki AL, Antropov VP (2017). Micromagnetic simulations with periodic boundary conditions: Hard-soft nanocomposites. J. Magn. Magn. Mater..

[11] Fangohr H, Bordignon G, Franchin M, Knittel A, de Groot PA, Fischbacher T (2009). A new approach to (quasi) periodic boundary conditions in micromagnetics: The macrogeometry. J. Appl. Phys..

[12] Fredkin D, Koehler T (1990). \Hybrid method for computing demagnetizing fields. IEEE Trans. Magn..

[13] Alnæs M, Blechta J, Hake J, Johansson A, Kehlet B, Logg A, Richardson C, Ring J, Rognes ME, Wells GN (2015). The fenics project version 1.5. Arch. Numer. Softw..

[14] Rathgeber F, Ham DA, Mitchell L, Lange M, Luporini F, McRae AT, Bercea G-T, Markall GR, Kelly PH (2016). Firedrake: Automating the finite element method by composing abstractions. ACM Trans. Math. Softw. (TOMS).

